# The Implementation and Evaluation of Individual Preference in Robot Facial Expression Based on Emotion Estimation Using Biological Signals

**DOI:** 10.3390/s21186322

**Published:** 2021-09-21

**Authors:** Peeraya Sripian, Muhammad Nur Adilin Mohd Anuardi, Jiawei Yu, Midori Sugaya

**Affiliations:** College of Engineering, Shibaura Institute of Technology, Tokyo 135-8548, Japan; nb17106@shibaura-it.ac.jp (M.N.A.M.A.); al17125@shibaura-it.ac.jp (J.Y.); doly@shibaura-it.ac.jp (M.S.)

**Keywords:** robot, robot facial expression, emotion estimation, biological signals, EEG, HRV, affect analysis, human-robot interaction, virtual agent

## Abstract

Recently, robot services have been widely applied in many fields. To provide optimum service, it is essential to maintain good acceptance of the robot for more effective interaction with users. Previously, we attempted to implement facial expressions by synchronizing an estimated human emotion on the face of a robot. The results revealed that the robot could present different perceptions according to individual preferences. In this study, we considered individual differences to improve the acceptance of the robot by changing the robot’s expression according to the emotion of its interacting partner. The emotion was estimated using biological signals, and the robot changed its expression according to three conditions: synchronized with the estimated emotion, inversely synchronized, and a funny expression. During the experiment, the participants provided feedback regarding the robot’s expression by choosing whether they “like” or “dislike” the expression. We investigated individual differences in the acceptance of the robot expression using the Semantic Differential scale method. In addition, logistic regression was used to create a classification model by considering individual differences based on the biological data and feedback from each participant. We found that the robot expression based on inverse synchronization when the participants felt a negative emotion could result in impression differences among individuals. Then, the robot’s expression was determined based on the classification model, and the Semantic Differential scale on the impression of the robot was compared with the three conditions. Overall, we found that the participants were most accepting when the robot expression was calculated using the proposed personalized method.

## 1. Introduction

In recent years, the use of robot services has expanded in commerce and welfare facilities due to the declining birthrate and aging society [1]. In situations that require human–robot communication, such as communication with older adults in welfare facilities, communication with customers in commercial settings, or other services that require communicate with robots, it is necessary for the robot to maintain a good impression. The impression, or acceptance, of a robot can be enhanced through facial expression, nonverbal expression, voices, etc. [2].

To enhance human–robot interaction, the robot must be able to recognize, interpret, and respond effectively to the human’s social cues. That is, in human–human communication, a person often displays his/her emotion through conscious and unconscious means, such as facial expression and nonverbal behavior [3]. A robot that can interpret such information will be able to respond and assist more effectively based on the human’s emotion [4]. This would result in more effective and engaging interactions with users, which would lead to better acceptance of these robots [5]. It is now possible to automatically detect such affect information via facial expressions [6,7,8], body language [8,9], voice [6,8] or a combination of facial expressions and voice recognition [6], and biological signals [10,11].

In this study, we focused on improving the acceptance of the robot’s facial expression for better human–robot communication by changing the robot expression according to the emotion information of its interacting partner. We did not consider any specific situation for the human–robot communication in our experiment. We hypothesized that some people may prefer when the robot expresses similar emotions, whereas others may not. Therefore, we investigated whether individual differences affect the personal preferences for robot expression. To clarify our hypotheses, we developed a machine learning model to define the relationship between personal preference and emotion estimated from the biological information. Accordingly, we developed a logistic regression model that predicts the personal preference for robot facial expression. As a result, it was found in the experiment that our proposed personalization method is effective for improving the acceptance of the robot facial expression based on the increase in the impression rating score and the increase in the frequency of “Like” clicked during the experiment.

## 2. Background

Several studies have investigated the emotion estimation function of robots for better human–robot communication. Some studies have estimated human emotions using biological signals analysis methods, such as voice recognition and facial expression recognition. A study by Yamano et al. performed an emotion estimation from the human voice using emotional voice analysis software. The estimated emotion was used to determine the robot facial expression in order to investigate people’s acceptance of the robot based on the emotion synchronization effect. The results of the study suggested that emotional synchronization boosted the positive impression of the robot [12]. In addition, Li et al. used facial expression recognition in their study of the emotional synchronization effect for human–robot communication. They compared the impressions of the robot between the synchronization and non-synchronization of the emotion, which was estimated from the participants using facial expression recognition. They found that emotional synchronization-based communication resulted in better impressions of the robot compared to non-synchronization, thus increasing people’s willingness to communicate with the robot in a favorable manner [13].

Jimenez et al. associated a learning-supported robot with an emotional expression. A model was proposed in which the robot facial expression changed according to the correct response for a given task and the time spent while studying. This model allowed the robot to synchronize the emotion with the participants. The learning effect and intimacy between the robot and participants were examined. As a result, it was suggested that the synchronized emotional expression of the robot may improve the performance of the participants, and tend to generate intimacy between the robot and participants [14]. Another study by Tanizaki et al. [15] also implemented a learning-supported robot that was able to express emotions. The participants in this experiment undertook the Synthetic Personality Inventory (SPI) test. The SPI test is a multiple-choice aptitude test that is usually undertaken by graduates in Japan as part of the process of finding employment. In the experiment, the robot expressed emotions, depending on the participants’ answers on the test, by manipulating its facial expressions and gestures, such as arm movements, to show a happy emotion. As a result, it was suggested that the combination of facial expression and physical movements could give the participants a favorable impression of intimacy and kindness.

However, the emotion estimation methods used in previous studies were solely based on controllable expressions. Face expression and voice intonation can be controlled, and may thus conceal the real emotion. Therefore, Kurono et al. [16,17] compared the impression of the robot based on the emotion estimated from controllable and uncontrollable expressions. The controllable expressions were derived from facial expression recognition, whereas the uncontrollable expressions were analyzed from brain waves and heart rate measurement. Brain waves and heart rate measurements may indicate an involuntary activity that usually occurs unpredictably. The authors measured the biological signals within a certain period of time to determine the estimated emotion and presented an image of the facial expression to be used as the robot expression. Then, the estimated emotions from controllable and uncontrollable expressions were compared. As a result, it was suggested that the emotion synchronized with the uncontrollable expression, rather than the controllable expression, provided participants with an impression of the robot’s intelligence.

In studies by Kurono et al. [16,17], the authors compared the impression rating of robot facial expression, which was synchronized from the emotion of the participant. In their work, the emotion was estimated from facial expression and biological signals (heart rate and brain waves). They found that the emotion estimated from biological signals, which was derived from uncontrollable expressions, resulted in a high concordance rate. Subsequently, Kajihara et al. [18] expanded the work to determine a suitable synchronization method for the robot expression and the emotion estimation approach of uncontrollable expressions. In Kajihara et al.’s later work [19], they compared the participant impression rating of the robot facial expression under two conditions: synchronized and inversely synchronized with the estimated emotion. The synchronized condition is the traditional version, in which the robot shows an expression similar to the estimated emotion. For the inversely synchronized condition, the robot shows the opposite emotion (based on Russel’s circumplex model of affect) to the estimated emotion. The results suggested that participants can develop the impression of intimacy and other aspects of the robot through emotion synchronization, but individual differences can affect the impression. They specifically noted that the interpretation of reverse synchronization differs individually. For example, some participants found it undesirable when the robot showed a happy expression when the participant was feeling sad, whereas others found it to be encouraging. It can be implied that people have different impressions of the same robot’s facial expression. Therefore, these individual differences must be taken into account when evaluating the robot’s facial expression.

In this study, we aimed to investigate a method to obtain a positive impression of the robot’s expression by considering individual differences. For this purpose, we validated the following factors in a preliminary experiment: 1. The type of robot facial expression; and 2. conditions for selecting the robot’s facial expression. Then, we evaluated the individual differences regarding the impression of the robot’s expression by applying a machine learning method for individual preference classification based on biological signals.

## 3. Proposed Method

Kajihara et al. suggested that real-time emotion estimation at an interval of 2.5 s produced the highest rating of the impression of a robot [18,19]. Therefore, in this research, we used the approach proposed by Kajihara et al. to estimate the emotions using biological signals, and thus regularly determine the robot facial expressions under three conditions: synchronize, reverse synchronize, and funny facial expressions. We used brain waves and the heart rate, for which changes cannot be controlled and are arbitrary, for the measurement of biological signals used to estimate the emotions. The robot facial expression is displayed for a short period in different conditions based on the estimated emotions. Then, the participants answered a subjective evaluation questionnaire to determine the individual differences in the impression of the robot. The relationship between the measured biological signals and the answers to the subjective evaluation questionnaire was investigated.

### 3.1. Emotion Estimation Method Using Biological Signals

In this study, the emotions were estimated from biological signals based on the brain wave and heart rate measurement analysis of Ikeda et al. [10]. Because the brain waves and heart rate can be measured continuously in real time [20,21,22], it is possible to change the robot facial expressions based on the estimated emotions by applying the methods from both Kajihara et al. [18,19] and Ikeda et al. [10]. If the robot is able to change and express emotions in real time during communication, the situation will more closely resemble human–human communication.

The emotion estimation methods in [10,18,19] correlate the values obtained by two physiological sensors, i.e., the brain waves (EEG) and heart rate variability (HRV), to the Arousal and Valence axes, respectively, in Russell’s circumplex model of affect [23]. The EEG can be used to measure the state of concentration and is reported to be negatively correlated with the subjectively evaluated level of arousal [24,25]. The HRV is a relatively reliable index for detecting stress and negative emotions [26]. Numerous studies have shown that the emotions estimated using these methods are closely related to the subjective evaluation results obtained in the experiments. These approaches have been used in numerous applications, such as social network feedback analysis, audience reaction analysis, and robot hospitality analysis [27,28,29,30].

For the EEG, we used the Attention and Meditation indexes provided by NeuroSky brainwave mobile™. The Attention and Meditation indexes are calculated on a relative scale of 0 to 100. For both indexes, the computation algorithms are based on both temporal and frequency domains. According to [31], the exact algorithm for computation has not been published, but the manufacturer indicates that the Attention index emphasizes beta waves and the Meditation index emphasizes alpha bands. Here, the arousal axis is defined as the difference between Attention and Meditation (Attention – Meditation). The valence axis is defined using the pNN50, which can be calculated from HRV. The pNN50 is the index typically used to measure the state of autonomic nerves via measurement of the valence level from the vagal tone intensity [32]. The pNN50 shows the rate at which the difference between the 30 adjacent R-R intervals (RRI) is greater than 50 milli seconds.

**Figure 1:**
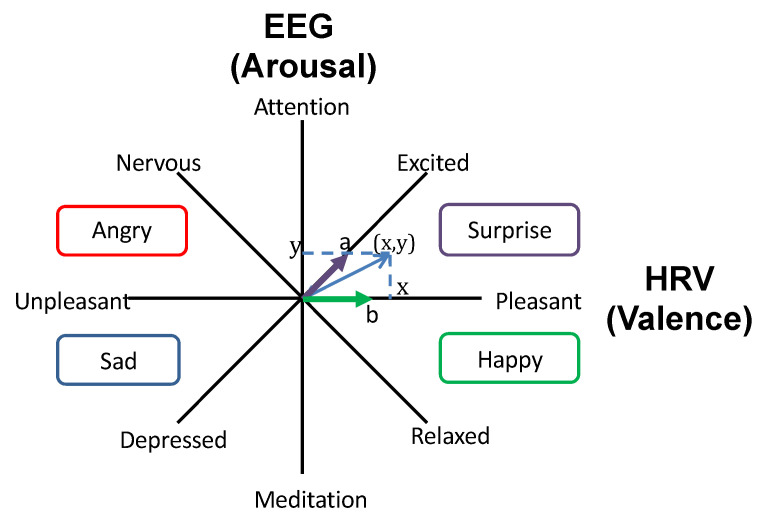
The mapping of EEG (*y*-axis) and HRV (*x*-axis) values on arousal (vertical axis) and valence (horizontal axis) in Russell’s circumplex model of affect to estimate emotion.

Each measured index can be plotted as shown in Figure 1, which is based on Russell’s circumplex model to classify the emotion [23]. The *y*-axis shows the arousal level, whereas the *x*-axis represents the valence level.

### 3.2. Robot Facial Expression

The study of Kajihara et al. examined four types of robot facial expression based on the four basic emotions [18]. In addition to the four basic emotions, we added another type of expression to clarify the individual differences in the impression of facial expressions. According to the article by Akita Mami [33], the bright and cheerful expression obtained the highest rating, of around 37%, as the expression that improved people’s first impression. Therefore, the “funny expression” was added as the representative of the bright and cheerful expression.

**Figure 2:**
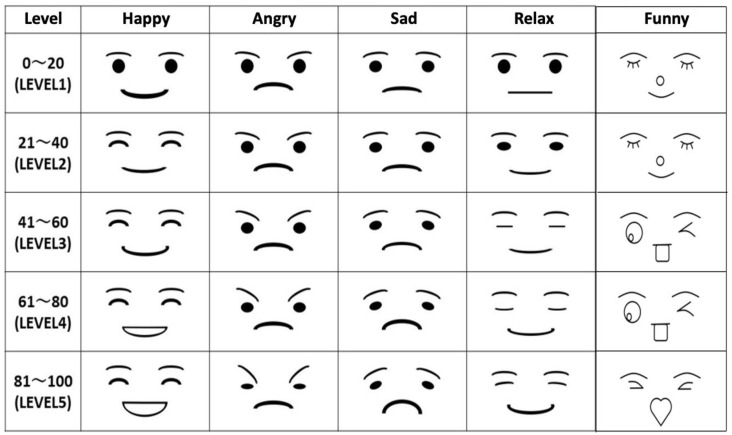
Robot expression used in our work.

Because the “funny expression” may be favored or rejected, this kind of expression can be used to investigate individual differences. Figure 2 shows five robot facial expressions used in this work: happy, angry, sad, relaxed, and funny. For each type of expression, there are five levels of emotional intensity. These are calculated from the emotion values obtained from the length to point x,y in Figure 1 based on Kajihara et al.’s approach [18,19]. The higher the emotional values, the higher the emotional intensity, as shown in Figure 2.

To determine the robot’s facial expression, Kajihara et al. investigated the method for synchronizing the robot’s expression with the estimated emotion. They found that the best impression was achieved when the robot’s facial expression was synchronized with the estimated emotion calculated cumulatively at an interval of 2.5 s.

### 3.3. Conditions for Selecting Robot Expression

The robot facial expression was determined differently according to the following three conditions:

1. Synchronized—robot expression is the same as the estimated emotion;
2. Inversely synchronized—robot expression is opposite to the estimated emotion;
3. Funny expression—the robot displays a funny expression regardless of the estimated emotion.

**Figure 3:**
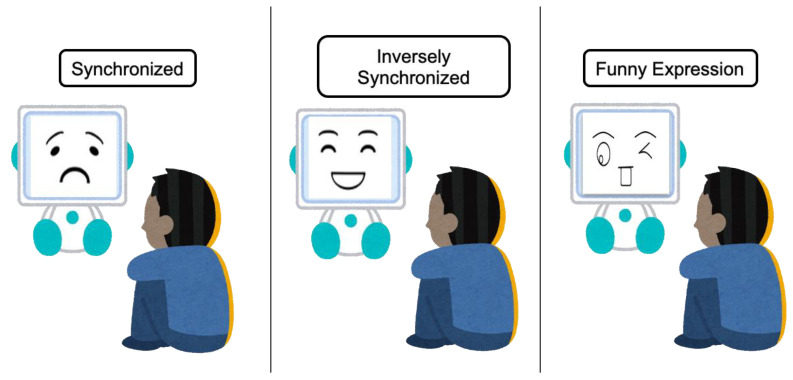
Three conditions for determining robot facial expression.

Figure 3 shows the simulation for all conditions. In the figure, the participant is feeling sad. In the synchronized condition, the robot shows a sad expression, whereas it shows a happy expression in the inversely synchronized condition. Finally, in the case of the funny condition, the robot expresses a funny facial expression regardless of the estimated emotion. In addition, emotion intensity is derived from the estimated emotion for all conditions.

## 4. Preliminary Experiment

The purpose of the preliminary experiment was to investigate individual differences in the impression of the robot’s expression. The participant was required to wear an EEG and a pulse sensor throughout the experiment. In each trial, the participant listened to music that evoked a happy or sad emotion. For later analysis of the individual preferences, the participant was asked to provide feedback regarding the impression of the robot, according to the estimated emotion in each of the three experimental conditions: synchronized, inversely synchronized, and funny. The order of the conditions was random.

### 4.1. Participant

Three male students (age range 18–25) voluntarily participated in the experiment with signed consent.

### 4.2. Stimuli

The stimulus used in the experiment was an emotive music clip with a length of 75 s. Two types of emotion-evoking music were prepared: happy and sad. The music clips were created by combining four music clips selected from the emotion-evoking music database [34] of the Finnish Center of Excellence in Interdisciplinary Music Research at the University of Jyvaskyla. The database was constructed from the study of music-mediated emotions. This database contains 110 film soundtracks, each of which is approximately 15 s long. All music is categorized by professional musicians based on the dimensional and discrete emotion model into several emotions, such as valence, energy, tension, anger, fear, happy, sad, beauty, etc. For each quadrant of the Arousal–Valence space model, we selected two songs based on the highest scores for the corresponding emotions. From this database, we selected the top ten music clips in the “happiness” and “sadness” category to create happy and sad emotion-evoking music clips, respectively.

### 4.3. Procedure

The participants were asked to wear an EEG, pulse sensor, and earphones during the whole experiment. A monitor to show the robot’s expression was set in front of the participant. After confirming that all data was being retrieved from all sensors, the experimental procedure for the participant was as follows:

1. Stay still (rest) for 60 s for a baseline measurement.
2. Listen to the 75 s emotion-evoking music clip. At the same time, the monitor showed a robot’s expression. While listening to the music, the participant could click the mouse to indicate whether he/she liked or disliked the robot expression at any time.
3. Rest for 60 s.
4. Repeat steps 2 and 3 for the three experimental conditions for happy and sad stimuli, respectively.

### 4.4. Results

**Figure 4:**
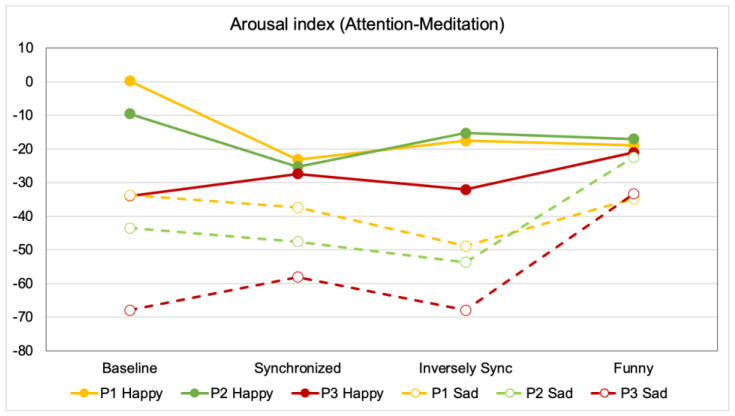
Comparison of average EEG (arousal) values for all participants. (P1 Happy: The value when participant 1 listened to happy music).

Figure 4 shows the comparison of average EEG values (calculated from the difference of Attention and Meditation) for each experiment condition, while being exposed to happy and sad stimuli. Each participant’s data is shown using different colors. The solid and dotted lines show the data obtained when the participant listened to happy and sad music, respectively. The positive value shows that the Attention value is higher than the Meditation value (Attention predominates), whereas the negative value shows that the Meditation value is higher than the Attention value (Meditation predominates). From the figure, it is interesting to note that the changes in EEG in the inversely synchronized condition and funny expression are large when the participants listened to sad music, but small or unnoticeable when the participants listened to happy music, compared to the baseline.

**Figure 5:**
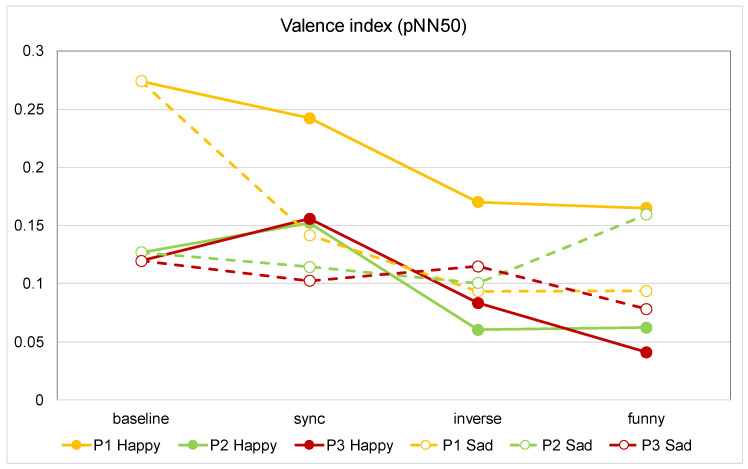
Comparison of average HRV (valence) values for all participants. (P1 Happy: The value when participant 1 listened to happy music).

Figure 5 shows the comparison of the average valence (pNN50) value between the baseline and the conditions of synchronized, inversely synchronized, and funny expression for all participants. The figure shows that valence was highest in the synchronized condition for all participants when listening to happy music. While listening to happy music, all participants’ valence tended to decrease when the expression was changed from synchronized to inversely synchronized. Furthermore, the changes were very small when the participants listened to sad music. It can be seen from the figure that valence was highest at the different conditions for each participant, thus showing individual differences when exposed to the sad stimulus.

### 4.5. Discussion and Points to Be Improved

From the result, it can be inferred that the biological signals showed individual differences in the robot’s facial expressions. The implications of the result are summarized as follows:

1. When the participant has a positive emotion, a robot showing a synchronized emotion can create pleasure.
2. When the participant has a negative emotion, the good impression of the robot’s expression varies depending on the individual.

In this experiment, the valence values were often low for all three participants, so the participants were likely feeling frustrated or bored during the experiment. In addition, the results of the subjective evaluation may not be consistent with the results of the biological signal data (e.g., I like the robot’s facial expression, but the valence value is low). Thus, further consideration is required of the experimental environment.

## 5. Analysis of Individual Preference Using Machine Learning

As confirmed in the results presented above, the biological information when a subject looks at the robot’s facial expressions differs from person to person. We hypothesized that if the robot’s facial expression can be synchronized with human emotions, human emotions will not be positive; rather, the expression will be positive or negative depending on the person’s emotions and other factors.

We thought it necessary to model and discuss the features that affect the emotions of human–robot facial expressions. For this purpose, we aimed to identify the complex reactions of these people using biological information to represent the features of the individual differences, which are based on personal preferences. To clarify this, we constructed a model from the features using a simple supervised machine learning method.

First, we constructed a simple preference model that reflected the biological information. To construct this model, it was necessary to collect data by executing an experiment. The experiment was constructed such that a person looked at a robot’s facial expression, and was asked to provide feedback in real time about whether he/she “liked” or “disliked” the robot’s facial expression. The robot facial expression changed based on the participant’s emotion. Because the features of the biological information were changed dynamically according to the stimulation, we collected the feedback from mouse clicks as the personal preference data. The personal preference data was used as the response variable, and the unconscious biological, arousal, and valence data were used as the explanatory variables, in the training data. To achieve this, two procedures were undertaken, as explained in the following sub-sections: Model and Data Collection.

### 5.1. Model

We made assumptions about the relationships between the participant’s personal preference data (feedback from mouse click) and his/her biological data (EEG and HRV) used in this research; the model was required to express the relationship between the personal preference data and multiple biological parameters. If these are simple linear relationships, multiple regression analysis is generally used to explain the objective variable with multiple explanatory variables. However, because it is assumed that people’s preference for evaluating the facial expression of a robot will be predicted, we used logistic regression. Logistic regression can be used to stochastically predict the objective variable, it is relatively simple, and the relationship between features is easy to understand.

Logistic regression is expressed by the following equation, where l is the logit of probability p:

(1) $$l=\left(\frac{p}{1-p}\right)\;=\beta_{0}+\beta_{1}x_{1}+\cdots +\beta_{p}x_{p},$$

(2) $$p=(1/\left(1+exp\left(-l\right)\right)\;=\;\left(\frac{1}{1+exp\left[-\left(\beta_{0}+\beta_{1}x_{1}+\cdots +\beta_{p}x_{p}\right)\right])}\right),$$

where p is the objective variable represented by the probability, $\beta_{0}$ is a constant, and $\beta_{0}-\beta_{p}$ is the partial regression coefficient. Here, we use p that predict the “like” and “dislike” feedback. The explanatory variables used here are the EEG and the HRV values (PNN50), which correspond to the two indices (arousal and valence) used to classify emotions using biometric information.

### 5.2. Data Collection

The datasets were prepared for the construction of the personal preference model via machine learning, as explained previously. This sub-section describes the method used for preparing the dataset for the machine learning model using the data collected from the experiment. The experiment methods, including the procedure and selection of stimuli, are described in the preliminary experiment section.

From the experiment, we determined sections (start/stop timestamps) when each music stimulus was presented to collect EEG and HRV data for the analysis. Because the EEG sensor and pulse sensor were unsynchronized, the data were obtained from the most recent EEG and HRV data, each second. Because each of the music stimuli was presented for about 75 s, approximately 75 entries of EEG and HRV were generated for music clip. Subjective evaluation results were also assigned to the physiological data of the corresponding music. Finally, we constructed a dataset as an input for machine learning-based classification models using EEG and HRV indexes as input features.

**Figure 6:**
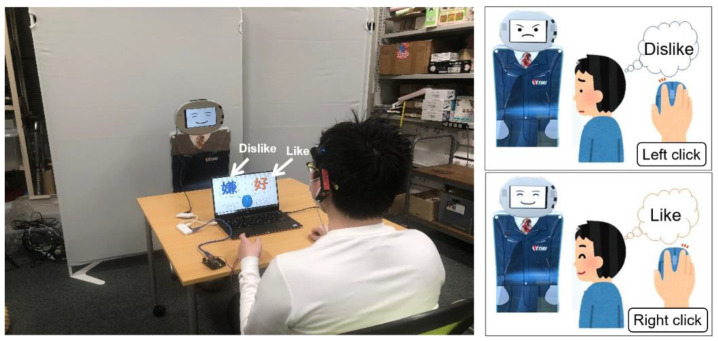
A photo of the experiment, in which the participant clicked the left or right mouse button to indicate “dislike” or “like”, respectively.

During the experiment, the participant was instructed to freely provide feedback regarding the robot expression. The participant was able to provide the feedback by clicking the left or right button on the mouse, indicating “dislike” or “like”, respectively (Figure 6). Here, the biological data used in the model was captured for 30 s before the first mouse click. For the target variable of the logistic regression, we utilized mouse click patterns from the subjective evaluation data, where the value of 1 indicates “like”, and 0 indicates “dislike”. Here, we utilized the equation to identify the biological data obtained when the subjective evaluation indicated “like” or “dislike” for each subject. Then, we plotted the distribution of the biological data for each subjective evaluation to classify the subjects’ individual preferences.

### 5.3. Model Accuracy

In this study, we verified the classification model by computing the model accuracy, ACC, as follows,

ACC = (TP + TN)/(TP + FP + FN + TN)(3)

where TP is True Positive, TN is True Negative, FP is False Positive, and FN is False Negative.

### 5.4. Fitting the Model to Data from Preliminary Experiment

**Figure 7:**
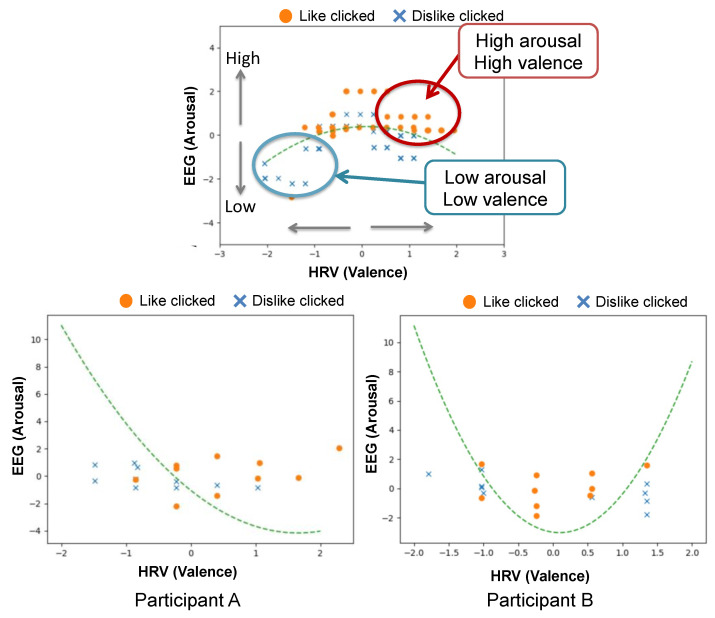
Classification model of Participant (**A**,**B**) (bottom left and bottom right figures). The fitted model for each participant is shown by the green dotted line. We illustrate how to interpret the results using the plot in the top middle figure.

Figure 7 shows the results of the classification model applied to the data of two participant (A and B) from the preliminary experiment. As shown in the figure, from the model of Participant A, “like” was correctly classified eight times (the number of orange circles on the right-hand side of the green dotted line) and incorrectly classified two times (the number of orange circles on the left-hand side of the green dotted line). By comparison “dislike” was correctly classified eight times and incorrectly classified two times. A similar result was obtained from the data of Participant B. Therefore, we can conclude that, for both participants, an accuracy of 80% was obtained using our method.

From the EEG (arousal) and HRV (valence) value distribution obtained when the subjects clicked like/dislike in response to the robot facial expression, as shown in Figure 7, it can be seen that Participant A’s HRV showed a positive value when he/she clicked like, which shows the preferences of Participant A. By comparison, both Participant B’s EEG and HRV were in the middle range when he/she clicked like for the robot facial expression. From these observations, we can infer that individual differences can be clearly indicated by the distribution of the EEG and HRV values of the biological data based on the preferences of the classification model.

## 6. Main Experiment

The purpose of this experiment was to evaluate the individual differences in the impression of the robot expression in terms of a subjective evaluation. Here, we only used sad music as the emotion-evoking stimulus because, based on the preliminary experiment, sad music is more prone to individual differences. The subjective evaluation using a questionnaire in the seven scales of semantic differential (SD) format was conducted at the end of each condition (synchronized, inversely synchronized, and funny).

### 6.1. Participant

Ten volunteers (six males and four females, age range 18–35 years) participated in the experiment with signed consent.

### 6.2. Stimulus

The stimulus used in the experiment was a 75 s sad emotion-evoking music clip. The music was selected and created in the same manner as the stimuli used in the preliminary experiment.

### 6.3. Subjective Evaluation

**Figure 8:**
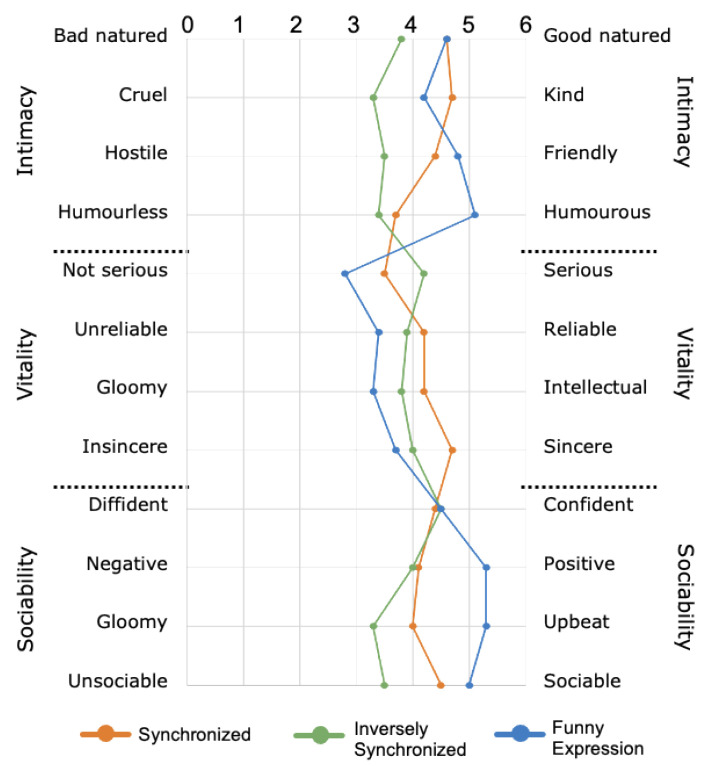
Comparison of impression rating of robot expression under three conditions (N = 10).

To evaluate the participant’s impression of the robot expression, we utilized the 12 adjective pairs that comprise the Japanese property-based adjective measurement method [35]. The impression rating on adjective pairs is undertaken using Osgood’s Semantic Differential (SD) [36] method. We defined the three attributes as “intimacy”, “sociability”, and “vitality”, and selected four corresponding property-based adjectives for each attribute, as shown in Figure 8. Finally, a total of 12 property-based adjectives were evaluated using the seven-rating scale.

### 6.4. Procedure

The method for conducting the experiment was similar to that of the preliminary experiment presented in Section 4.3. The experiment procedure is as follows:

1. Baseline measurement for 60 s.
2. The participant listened to a 75 s music clip while looking at the robot’s expression, which changed in real time. During this step, the participant could freely click “Like” or “Dislike”.
3. Answer the subjective evaluation questionnaire.
4. Rest for 60 s.
5. Repeat steps 2 to 4 for all three conditions.

### 6.5. Result and Discussion

#### 6.5.1. Impression Rating Score

Figure 8 shows the impression rating score result for each item. It can be seen from the figure that, when the robot expression is in the funny expression condition (blue line), the rating scores for all items in the sociability group were highest. By comparison, the rating scores for three of the four items in the vitality group were highest when the robot expression was in the synchronized condition (orange line).

From these results, it can be inferred that the effect of encouragement can result in impression differences. Regarding the reason that sociability resulted in a lower impression, it can be considered that this may be due to the effect of the “sarcasm” perception, which differs for each individual.

#### 6.5.2. PCA on Impression Rating Score

**Figure 9:**
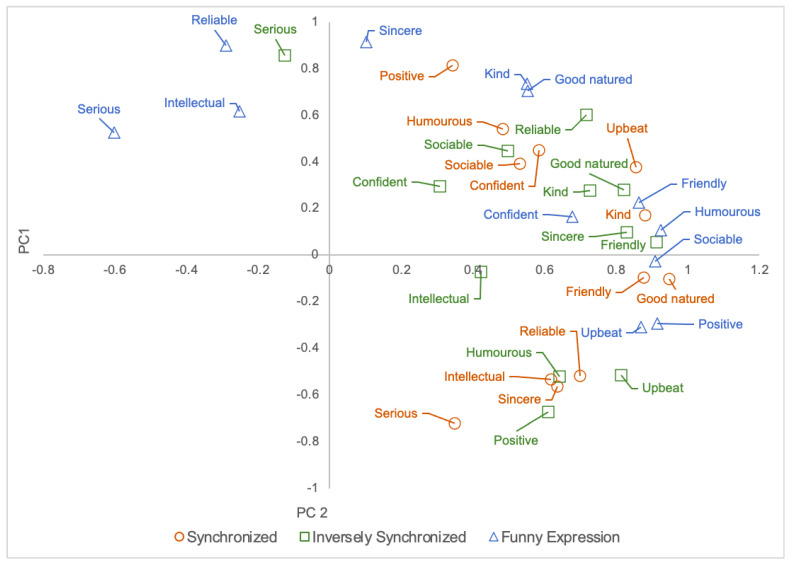
PCA.

A principal component analysis (PCA) was performed on the impression rating score of the robot expression. Figure 9 shows the PCA on the impression score for all three conditions. For the synchronized condition (Figure 9, circle), it can be seen that all items have positive factor loadings for the first principal component. This suggests that the first principal component is an index for evaluating the robot impression of all of the evaluation items. In the second principal component, all items in the vitality group had positive factor loadings. By comparison, among the sociability items, the item “serious” showed a particularly strong negative factor loading. This suggests that the second principal component influences the vitality-seriousness rating when the robot makes facial expressions that are synchronized with negative emotions.

For the inversely synchronized condition (Figure 9, square), the first principal component shows that all of the social attributes, with the exception of “serious”, had positive factor loadings. This also suggests that the first principal component is an index for evaluating the robot’s impression of the entire set of evaluation items. For the second principal component, the positive factor loadings are “good-natured”, “kind”, and “friendly” in the intimacy attribute; “serious”, “reliable”, and “sincere” in the sociability attribute; and “confident” and “sociable” in the activity items. This suggests that the second principal component affects the evaluation of positive emotions and seriousness when the robot expresses an emotion opposite to the participant’s negative emotions.

In the funny expression condition (Figure 9, triangle), all of the social items, with the exception of “serious” and “intelligent”, had positive factor loadings for the first principal components. From the figure, “humorous” showed a strong positive factor loading. In contrast, “serious” showed a particularly strong negative factor loading. This suggests that the first principal component affects the evaluation of humor-seriousness when the robot makes funny facial expressions. The second principal component showed strong positive factor loadings for all of the sociability attributes. However, negative factor loadings were found for all of the activity items, with the exception of “positive”, “upbeat”, and “sociable”. This suggests that the second principal component influences the evaluation of sociability-vitality when the robot makes funny expressions.

#### 6.5.3. Classification Model

**Figure 10:**
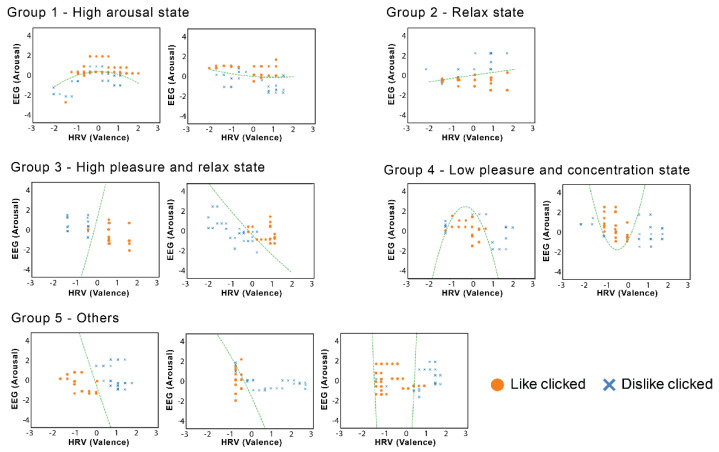
Grouping of participants based on the classification model. Each participant’s data is shown with the fitted model (green dotted line) in each graph.

By fitting the data to a logistic regression model based on the method discussed in Section 5, it is possible to divide the results of the ten participants from the main experiment, based on the relationship of their biological signals and their mouse click behavior, into five groups, as shown in Figure 10. It can be observed that the participants in each group tended to click “like” for the robot expression when their biological signals were as follows:

- Group 1—High arousal state, regardless of pNN50. This indicates that the participants may be concentrating and nervous, which led to excitement.
- Group 2—Relaxed state, regardless of pNN50.
- Group 3—High pleasure and relaxed state.
- Group 4—Low pleasure and concentration state.
- Group 5—Others; most of the observed models indicated the middle range of pleasure.

The participants in group 1 and 4 were found to prefer the feeling of excitement, whereas those of group 2 and 3 appeared to prefer feeling more relaxed. The data in group 5 most clearly showed the effects of individualism, and it was not possible to simply group these participants.

The results of the classification model based on the machine learning of the biological signals show that the influence of personality is significant. This confirms that there were individual differences in the perception of robot expression under the three conditions. Therefore, it is important to understand and analyze the individual differences to effectively enhance human–robot interactions via the manipulation of robot expression.

## 7. Follow-Up Experiment

We conducted a follow-up experiment with the same participants. In this experiment, the participants listened to sad music for 75 s to evoke emotions. The goal of this follow-up experiment was to clarify the difference in the impressions given by the robot’s facial expressions with and without the personalization function. Here, we analyzed and compared the impressions from each of the three conditions (synchronized, inversely synchronized, funny) with the personalized condition, which was built using the logistic regression from the main experiment. We used the robot’s facial expression obtained when the user clicked the “Like” button in the main experiment.

### 7.1. Selection of Robot Expression

The robot expression was selected based on the personalization function, which was analyzed from the classification model, as discussed in the main experiment’s results. The following points were considered for the personalization function:

1. The expression condition (synchronized, inversely synchronized, or funny expression) that yielded the highest average pNN50 value;
2. The time when the participant clicked “Like” was taken into account;
3. The robot expression at that timing was selected.

### 7.2. Participants

The participants for this experiment were the same individuals who participated in the main experiment. However, two of the participants could not participate in this follow-up experiment due to personal reasons. Therefore, the total number of participants was eight (six males, two females).

### 7.3. Experiment Method

In this follow-up experiment, the participant listened to the same sad emotion-evoking music that was used in the main experiment The robot expression was selected differently for each participant, as explained in Section 7.1. The subjective evaluation method and experiment procedure were similar to those of the main experiment, as explained in Section 6.3 and Section 6.4.

### 7.4. Results and Discussion

To clarify the impression effect of robot facial expressions for individuals, we compared the impression rating scores and the frequency of “Like” and “Dislike” clicks under four experiment conditions:

1. Synchronized;
2. Inversely Synchronized;
3. Funny;
4. Personalized.

The impression rating scores and frequency of “Like” and “Dislike” clicks of the first three experiment conditions were obtained from the main experiment. For condition 4 (personalized), we obtained the impression rating scores and the frequency of “Like” and “Dislike” clicks from the follow-up experiment.

#### 7.4.1. Impression Rating Scores

**Figure 11:**
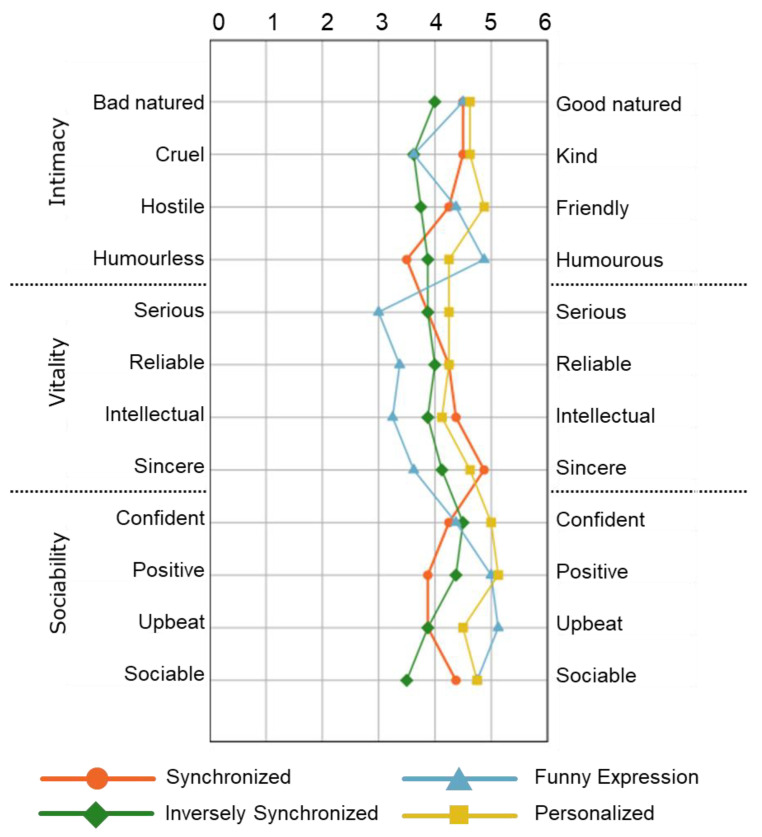
Comparison of impression rating toward robot expression under four conditions (N = 8).

The impression rating score results for each item are shown in Figure 11. The figure shows that the scores from personalized expression were highest among all expression conditions in most items in the intimacy and sociability groups.

It can be inferred that, when the robot expression is shown using our proposed personalized method, the participants felt more positive in terms of intimacy and sociability towards the robot expression. However, the personalized expression did not affect the impression of the vitality group.

#### 7.4.2. Analysis of the Frequency of Clicking “Like” and “Dislike”

**Figure 12:**
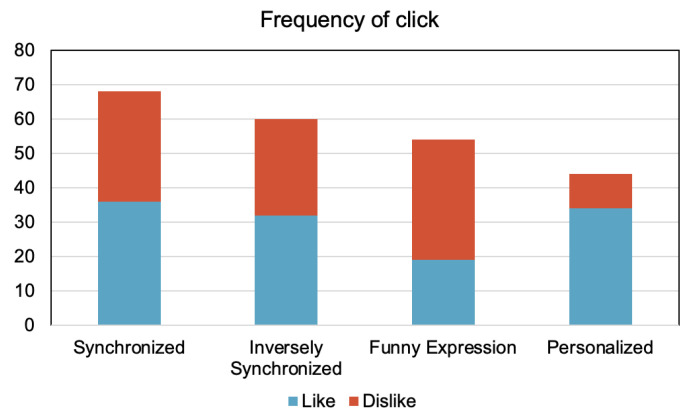
Frequency of clicking for each robot expression during the experiment.

We also analyzed the frequency of clicking the “Like” or “Dislike” button during the experiments. Because clicking “Like” or “Dislike” was able to be performed freely, the number of clicks for each participant varied; we counted the frequency of the total number of “Like” and “Dislike” clicks for each expression condition for the analysis. There was a significant association between the expression condition and whether the participant clicked “Like” or “Dislike”, X^2^(3) = 17.29, *p* < 0.0001. Figure 12 shows the frequency of clicks for each expression condition. It can be seen from the graph that the ratio for “Like” clicking (blue to red ratio) was the highest in the personalized condition.

## 8. Conclusions

The impression of the robot’s facial expression can be improved for better human–robot communication by changing the robot expression according to the emotional information of its interacting partner. We hypothesized that some people may prefer that the robot expresses an emotion similar to theirs, but some may not. In this work, we investigated whether such individual differences exist, and if they can be identified using physiological signals and machine learning. As a result, we proposed a method for personalization of robot expression based on the emotion estimated using an individual’s physiological signals.

HRV and EEG data were used to map the biological information onto Russel’s circumplex model of affect for emotion estimation. The result of a preliminary experiment showed that the participants felt pleasure when the robot facial expression showed a synchronized emotion during the positive stimulus. However, when the participant felt negative emotions, the impression of the robot’s expression varied depending on the individual. To investigate individual preference, we used a logistic regression algorithm to develop and evaluate a classification model based on biological data and feedback obtained during the experiment regarding the robot expression. The classification model achieved an accuracy of 80%, which suggested that this method can be used in determining the robot facial expression by considering individual preference. The main experiment was conducted to obtain biological and feedback data, in addition to a subjective evaluation using the Semantic Differential scale method. The analysis of the subjective evaluation showed that the effect of the inverse synchronization of emotion when the participant felt a negative emotion resulted in impression differences, which differed between individuals. Finally, we constructed a personalized method for selecting the robot facial expression based on the obtained data. The comparison of the subjective evaluation results across all conditions showed that the personalized condition achieved the highest impression rating in most items in the intimacy and sociability groups. In addition, the ratio of the frequency of “Like” clicks was the highest in the personalized condition.

To generalize our findings, we plan to evaluate our proposed method with a larger number of participants. Furthermore, we plan to continue the study in detail by creating the robot’s facial expression using an individual preferences model based on biological signals measured from the user in real time. Thus, this model may be applied in human–robot communication to improve robot services. Moreover, it is also essential to consider specific situations of human–robot interactions to acquire more details, and thus achieve improved robot service development.

## Data Availability

The data presented in this study are available on request from the corresponding author. The data are not publicly available due to the ethical declaration issue.
